# The Libel Suit of William Smith, Osteopathist

**Published:** 1898-12

**Authors:** 


### The Libel Suit of William Smith, Osteopathist.

---

*To the readers of The Medical Age:*

Dr. William Smith, Osteopathist, has a grievance against THE MEDICAL AGE, and demands $25,000 damages.

The ground of his plaint is an editorial, reflecting discredit on Dr. Smith, on the *Journal of Osteopathy,* and on osteopaths in general. The subject is set forth editorially in THE MEDICAL AGE of September 26, 1898, and a reprint of this editorial will be sent on application.

I need hardly assure any one familiar with the past record of the AGE, that William Smith, M. D., D. O., has a large contract on his hands. His quest for damages is likely to prove futile, and his armor will need patching if it is to withstand the hard legal knocks that will be showered and battered upon it before he touches one dollar of the AGE's money!

Pray do not fancy, however, that William Smith and Osteopathy are to be lightly dismissed with the contempt that they merit. There is no use in blinking the fact that the lack of efficient organization amongst reputable medical men has permitted the whole brood of quacks and charlatans to flourish apace. By the strangest irony of fate, Osteopathy, in some respects the most grotesque of medical aberrations, has well illustrated Lecky's dictum that a small but cohesive and determined minority can exert a political influence wholly disproportioned to its real weight and numbers

In Kentucky, thanks to the resolute leadership of a handful of physicians, ably guided by Dr. Mathews, the osteopaths have been driven from the State. Not so, in Missouri or—I blush to say it—in Michigan, Vermont, North Dakota, South Dakota, Illinois, Colorado, and North Carolina (*American Medico-Surgical Bulletin*).

In these more lax and indulgent communities Osteopathy boasts its numerous followers, its "schools of instruction," its periodicals of propaganda, its political influence in legislation, its shameful immunity from the penalties by which society properly seeks to rid itself of quackish parasites.

Emboldened by its success, Osteopathy now enters the courts and offers battle to a medical journal disputes its respectability. The challenge is accepted. In the interest of science, in defense of ethical and honorable medicine, in defiance of a quackery that constitutes a deep disgrace to an enlightened age and a stain on the communities which give it shelter, the AGE proposes to maintain its position and to continue its denunciations of the ignorant pretenders who fatten on the sufferings of the credulous and confiding.

Having put my hand to the plow in this uncompromising fight with quackery, I beg leave to assure you that there will be no turning back

I need not point out the bearings this contest must have on the interests of legitimate medicine, and I earnestly hope that the AGE may count on the moral support and commendation of the entire profession.

Faithfully yours,

WILLIAM M. WARREN,

Publisher Medical Age.

We reproduce the above from the *Medical Age,* October 1898, for the information of our readers. We repeat one line from the above and desire to emphasize it: *"There is no use blinking the fact that the lack of efficient organization amongst reputable medical men has permitted the whole brood of quacks and charlatans to flourish apace."* This JOURNAL has for years urged the profession of Texas to organize in every county. Not until that is done can there ever be unanimity of sentiment, nor can we ever influence legislation. The medical profession of Texas must speak as a unit, and the request for legislation to suppress the indiscriminate practice of medicine will be heeded.

We wish the *Age* success in its fight. Such suits result from the encouragement given "pathies" by the recognition of their pretentions by the law as "schools of medicine."

